# CTRP3 is a novel biomarker for diabetic retinopathy and inhibits HGHL-induced VCAM-1 expression in an AMPK-dependent manner

**DOI:** 10.1371/journal.pone.0178253

**Published:** 2017-06-20

**Authors:** Zheyi Yan, Jianli Zhao, Lu Gan, Yanqing Zhang, Rui Guo, Xiaoming Cao, Wayne Bond Lau, Xin Ma, Yajing Wang

**Affiliations:** 1 Department of Ophthalmology, The First Affiliated Hospital, Shanxi Medical University, Taiyuan, Shanxi, China; 2 Department of Emergency Medicine, Thomas Jefferson University, Philadelphia, PA, United States of America; 3 Department of Anesthesiology, The First Affiliated Hospital, Shanxi Medical University, Taiyuan, Shanxi, China; 4 Department of Physiology, Shanxi Medical University, Taiyuan, Shanxi, China; 5 Department of Orthopedics, The Second Affiliated Hospital, Shanxi Medical University, Taiyuan, Shanxi, China; East Tennessee State University, UNITED STATES

## Abstract

**Objectives:**

Diabetic retinopathy (DR) is a severe complication of chronic diabetes. The C1q/TNF-related protein family (CTRPs) has been demonstrated to exert protective effects against obesity and atherosclerosis in animal studies. Heretofore, the association between circulating CTRPs and DR patients has been unexplored. In the current study, we attempt to define this association, as well as the effect of CTRPs upon DR pathophysiology.

**Design:**

The present investigation is a case control study that enrolled control subjects and type 2 diabetes mellitus (T2DM) patients diagnosed with DR. Serum CTRPs and sVACM-1 were determined by ELISA.

**Results:**

Serum CTRP3 and CTRP5 levels were markedly decreased in patients with T2DM compared to controls (p<0.05) and inversely associated with T2DM. Furthermore, mutivariate regression and ROC analysis revealed CTRP3 deficiency, not CTRP5, was associated with proliferative diabetic retinopathy (PDR). Spearman’s rank correlation assay demonstrated an inverse association between CTRP3 and sVCAM-1. Finally, exogenous CTRP3 administration attenuated high glucose high lipid (HGHL)-induced VCAM-1 production in an AMPK-dependent manner in cultured human retinal microvascular endothelial cells (HRMECs).

**Conclusion:**

CTRP3 may serve as a novel biomarker for DR severity. CTRP3 may represent a future novel therapeutic against DR, a common ocular complication of diabetes.

## Introduction

Diabetic retinopathy (DR) is a common and rehabilitating complication of chronic diabetes, with rapidly increasing worldwide incidence [1]. It is a leading cause of poor visual acuity and blindness [2]. Due to the lack of effective early detection strategies [3], approximately 95% of DR subjects are unable to avoid visual acuity loss by the time of diagnosis [4].

DR has two stages, namely non-proliferative (NPDR) and proliferative (PDR). NPDR is the early stage, and is characterized by visible damage to small retinal blood vessels which causes macular edema (central retinal fluid leakage), the most common mechanism of vision loss in diabetic retinopathy. Advanced NPDR leads to PDR, which is characterized by severe small retinal vessel damage and reduced retinal oxygenation. This in turns prompts neovascularization (new blood vessel formation), the hallmark of PDR. There currently exists no consistently effective means to inhibit DR progression [5], underlining the need for both early DR detection and treatment advances.

Secreted by adipose tissue, adipokines are proteins involved in the vascular proliferative responses of many chronic diseases [6, 7]. Evidence suggests that adipokines may serve as prognostic markers, particularly the C1q complement/TNF-related proteins (CTRPs) family. The well-known adipokine adiponectin has anti-diabetic effect, and is a predictor of diabetes [8]. CTRPs have metabolic effects similar to adiponectin (APN), particularly CTRP3 (also known as cartonectin, cartducin, and CORS-26)[9, 10]. CTRP3 is anti-inflammatory adipokine [9, 11–14], and its levels decrease in diabetes [15]. Whether CTRP3 may be a predictor of the well-known complications of diabetes is unknown. CTRP5 ameliorates palmitate-induced apoptosis and insulin resistance, enhancing fatty acid oxidation and insulin sensitivity[16, 17]. CTRP5 may have therapeutic potential in the management of type 2 diabetes mellitus. However, whether CTRP3 or CTRP5 have potential as a screening marker for DR has not been investigated.

Similar to the diabetic condition itself, the pathogenesis of DR involve low-grade, chronic inflammation [18, 19]. In the early stages of inflammation, cytokines release induces secretion of various adhesion molecules, including the vascular cell adhesion molecule-1(VCAM-1) and intercellular adhesion molecule (ICAM-1) [20]. The relationship between CTRPs and adhesion molecules in the context of DR has not been explored.

Therefore, the aims of this study were (1) to evaluate the circulating CTRP levels of healthy subjects and type 2 diabetes mellitus (T2DM) patients, with and without DR, (2) to determine the relationship of CTRPs and DR (3) attempt to define the underlying mechanisms for any observed protective effect.

## Materials and methods

### Participants and eligibility

Enrollment for this study at the outpatient clinics of Shanxi Medical University the First Affiliated Hospital commenced from April 2015 until May 2016. 120 total subjects were enrolled. This included 30 healthy control subjects, 34 T2DM patients without retinopathy, and 56 total DR patients (31 non-proliferative diabetic retinopathy, NPDR and 25 proliferative diabetic retinopathy, PDR). All study protocols were approved by the Ethics Committee of Shanxi Medical University. Written informed consent was obtained from all subjects prior to study inclusion. T2DM was diagnosed per American Diabetes Association criteria: a fasting blood glucose (FBG)≥126 mg/dl, or a 2 hour blood glucose (after standard oral glucose tolerance test) exceeding or equaling 200 mg/dl, or random (non-fasting) blood glucose ≥ 200 mg/dl or HbA1c>6.5% [21]. Exclusion criteria for this study were: type 1 diabetics, severe comorbidities such as congestive heart failure, liver disease, malignancy, inflammatory process, pregnancy, or any factor affecting body weight such as hyperthyroidism, corticosteroids, or contraceptives.

### Biochemical and hormonal analysis

All patient blood samples were obtained after overnight fast. HbA1c, total cholesterol, high-density lipoprotein (HDL) cholesterol, low-density lipoprotein (LDL) cholesterol, triglycerides (TG), total cholesterol (TCH), and glucose were determined by commercial kit via Hitachi 7600 biochemical automatic analyzer (Hitachi, Tokyo, Japan). The TCH/HDL and TG/HDL ratios were calculated as an indicator of ischemic heart disease mortality and morbidity [22, 23].

### Measurement of plasma CTRP3 and CTRP5

Plasma CTRP3 and CTRP5 levels were determined by commercial enzyme-linked immunosorbent assay (ELISA) kit (Cat, SK00082-07 for CTRP3; Cat, SK00594-06 for CTRP5, Aviscera Bioscience, Santa Clara, CA) per manufacturer’s instructions. The minimum detectable levels of CTRP3 and CTRP5 were 2ng/ml and 150 pg/ml (intra-assay coefficient of variability (CV) 4–6% and inter-assay CV was 8–10%.

### Measurement of plasma sVACM-1

Soluble vascular cell adhesion molecule-1(sVCAM-1, an inflammatory plasma marker) was measured by a commercial ELISA kit (Cat, EK0537 Bosterbio, Pleasanton, USA). The minimal detectable concentration of sVCAM-1 was <10pg/ml (intra-assay CV 2.7% and inter-assay CV 3.5–5%).

### Cell culture and treatments

Human retinal microvascular endothelial cells (HRMECs, passage 2–3) (Cat, cAP-0010, Angio-Proteomie, Boston, MA, USA) were plated on six-well plates and cultured at 37°C (5% CO_2_). Upon reaching 80% confluence, HRMECs were randomized to receive one of the following treatments: Normal glucose/normal lipid culture for 48 hours (control, 5.5 mM D-glucose+19.5 mM L-glucose) followed by vehicle or CTRP3 (Cat, 00082–01 for CTRP3; Cat, 00594–02 for CTRP5; Aviscera Bioscience, Santa Clara, CA) (3 μg/ml, Bacterially expressed) treatment; or high glucose (HG, 25 mM D-glucose)/high lipids (HL, 250 μM palmitates) [24] culture for 48 hours (HG/HL) followed by vehicle or CTRP3 treatment (0.3, 1, and 3μg/ml) for 15 minutes or 1 hour. The effect of HGHL upon CTRP3's stimulatory effect was determined by western analysis.

### Western blot analysis

HRMECs were harvested and lysed for 10 minutes in cold lysis buffer containing protease inhibitors (Cat, P8340, Sigma, St. Louis, MO). 50 μg total proteins were immunoblotted with primary antibodies against Serine^473^ phosphorylated Akt (RRID, AB_329825), total Akt (RRID, AB_915783), Threonine^172^ phosphorylated AMPK (RRID, AB_330330), total AMPK (RRID, AB_330331), HRP conjuncted anti-rabbit IgG (RRID, AB_2099233), HRP conjuncted anti-mouse IgG (RRID, AB_330924) and VCAM-1 (Cat, 13662) (all from Cell Signaling, Danvers, MA). The membrane was exposed with Super-Signal Reagent (Cat, 34080, Pierce, Illinois, USA), and imaged by a Bio-Rad ChemiDoc Touch station.

### Small interfering RNA transfection

siRNA duplex oligonucleotides were designed for AMP kinase-α1 (AMPK-α1) target sequences, silencing respective gene expression. HRMECs were transfected by siPORTER siRNA transfection kit (Cat, AM4503, Ambion) per manufacturer’s protocol with siRNA duplex against AMPK-α1 (5’-UGCCUACCAUCUCAUUAdTdT-3’) and universal control (Santa Cruz). Briefly, HRMECs were plated on six-well plates before transfection. After reaching 80% confluence, siRNA was applied to each well (final concentration 50 nM).

### Cell migration assays

To analyze HRMEC transwell migration activity, the upper surface of the transwell filters was coated with matrigel. Cells suspended in serum-free media were then added to the chamber, which was placed in a 24-well plate containing complete medium. After 24-hour incubation at 37°C, the filters were gently removed and matrigel on the upper surface of the filters was removed by cotton swabs. Cells on the underside of transwell filters were fixed with 4% paraformaldehyde for 30 minutes, stained with 0.1% crystal violet for 10 minutes, and photographed. Cell migration was quantified as the number of migrated cells in the treatment group divided by the number of migrated cells in the control group as described [25].

### Statistical analysis

Descriptive analysis was applied. Normality was tested by the Shapiro-Wilk test for all quantitative variables. Data with normal distribution are expressed as mean±SD. Skewed distributed variables are expressed with median±interquartile ranges (IQR). Differences between groups were tested using the Mann-Whitney U test for continuous variables. The Chi-square test evaluated for differences in the distribution of categorical variables. The effects of different variables were calculated using univariate logistics regression analysis.

We built a multivariate model among the significant variables noted by univariate analysis to determine potential screening markers for DR, after adjustment for other risk factors. The predicted probability of being diagnosed with DR was used as a surrogate marker to construct receiver operating characteristic (ROC) curves. The area under the ROC curve (AUC) served as an accuracy index evaluating the diagnostic performance of the noted marker. Spearman Rank correlation calculated the association between variables.

Western blot densities were measured via Image Lab 5.2 (Bio Rad, Hercules, CA). All statistical analyses were conducted with SPSS version 22.0 (SPSS, Chicago, IL, USA). P values less than 0.05 were considered significant.

## Results

### General characteristics

120 patients were enrolled in the current study (30 healthy control individuals and 90 patients with type 2 diabetes). The diabetic patients included 34 patients with no retinopathy (28.3% of total enrolled), 31 NPDR patients (25.8%) and 25 PDR patients (20.8%). Demographic data analysis revealed no significant difference amongst the four groups (Control, T2DM without retinopathy, NPDR, and PDR) with respect to gender, age, BMI, TG, TCH, TCH/HDL, TG/HDL (Table 1). However, concentrations of both CTRP3 and CTRP5 were significantly decreased in T2DM patients compared to control (p<0.001) and both CTRP3 and CTRP5 concentrations were lower in the PDR group compared to NPDR (p<0.05, Table 1, Table A in S1 File). sVCAM-1 concentrations were significantly increased in T2DM compared to control (p<0.001) and sVCAM-1 serum levels increased in PDR compared to NDFR (p<0.05, Table 1).

**Table 1:** General characteristics of study subjects.

Variable	Control subjects	T2DM without retinopathy	NPDR	PDR	p Values
**N (male %)**	**30 (0.6)**	**34 (0.53)**	**31 (0.58)**	**25 (0.44)**	**0.65**
**Age (years)**	**50.53±18.59**	**55.47±9.05**	**58.71±11.23**	**58.24±11.56**	**0.19**
**BMI (kg/m2)**	**23.37±2.96**	**22.77±3.01**	**24.16±3.99**	**23.99±2.59**	**0.22**
**Triglycerides (mmol/L)**	**4.73±0.77**	**4.94±1.21**	**4.69±1.31**	**4.88±1.12**	**0.69**
**TCH (mmol/L)**	**1.98±0.96**	**1.52±0.81**	**1.72±1.4**	**1.95±0.91**	**0.08**
**HDL-cholesterol (mmol/L)**	**1.08±0.22**	**1.36±0.73**	**1.12±0.20**	**1.18±0.3**	**0.26**
**LDL-cholesterol (mmol/L)**	**2.81±0.85**	**2.64±0.69**	**2.71±1.03**	**2.89±0.99**	**0.89**
**TCH/HDL**	**4.47±1.08**	**4.01±1.02**	**4.25±1.09**	**4.33±1.35**	**0.15**
**TG/HDL**	**2.06±1.56**	**1.44±0.94**	**1.56±1.22**	**1.75±1.13**	**0.54**
**Glucose (mmol/L)**	**4.84±0.74**	8.08±1.82**	9.60±2.31**	8.35±2.00**	**<0.001**
**HbA1C (%)**	**4.55±0.63**	8.82±2.76**	13.93±6.05**	12.51±6.53**	**<0.001**
**N of Hypertension (%)**	**4 (0.13)**	**7 (0.21)**	**5 (0.16)**	**7 (0.28)**	**0.34**
**N of Diabetic nephropathy (%)**	**0 (0)**	**0 (0)**	**7 (0.23)**	**15 (0.60)**	**<0.001**
**sVCAM-1 (pg/ml)**	**10055.47±1130.68**	14789.00±3486.57**	18362.45±4686.17**	23835.52±3061.85**^a^	**<0.001**
**CTRP3 (ng/ml)**	**187.25±51.91**	142.31±13.81**	120.92±30.69**	76.83±18.21**^a^	**<0.001**
**CTRP5 (ng/ml)**	**486.13±88.13**	288.04±20.64**	251.52±52.90**	231.59±39.46**^a^	**<0.001**

Data are expressed as mean± (SD) or n (%). p values were determined by using Mann-Whitney U test or Chi-square test as appropriate comparison.

BMI, body mass index; SBP, systolic blood pressure; DBP, diastolic blood pressure; TCH, total cholesterol; HDL, high-density lipoprotein; LDL, low-density lipoprotein; TG: triglycerides; HbA1c, glycated hemoglobin A1c; CTRP, C1q/TNF-related protein.

**p<0.01 Comparison with Control subjects.

a, p<0.05 Comparison between NPDR and PDR.

### CTRP3 is an independent predictor for DR

Univariate logistics regression analysis followed by multivariate analysis regarding DR and patient laboratory results are shown in Tables 2 and 3. Results showed that DR has no relationship to gender, BMI, Triglycerides, TCH, HDL, LDL, and hypertension but DR was positively correlated with glucose, HbA1C, sVCAM-1, CTRP3, and CTRP5 (Table 2 and Table B in S1 File). After adjustment for other risk factors, multivariate regression analysis demonstrated CTRP3 was inversely correlated with DR (p<0.05, Table 3 and Table C in S1 File). CTRP3 was an independent predictor for DR (hazard ratio, 1.04; 95% CI, 1.01–1.08; P = 0.01).

**Table 2:** Correlation between DR and laboratory characteristics.

Variable	Univariate Analysis		
	OR	95%Cl	P
**Age (years)**	1.03	(1.00–1.06)	0.03
**Gender**	0.78	(0.38–1.61)	0.51
**BMI (kg/m2)**	1.11	(0.99–1.25)	0.08
**Triglycerides (mmol/L)**	0.95	(0.68–1.31)	0.73
**TCH (mmol/L)**	1.08	(0.77–1.53)	0.65
**HDL-cholesterol (mmol/L)**	0.64	(0.26–1.60)	0.34
**LDL-cholesterol (mmol/L)**	1.06	(0.71–1.60)	0.77
**TCH/HDL**	0.94	(0.70–1.27)	0.70
**TG/HDL**	1.05	(0.76–1.45)	0.76
**Hypertension**	1.457	(0.59–3.58)	0.412
**Glucose (mmol/L)**	1.48**	(1.27–1.72)	<0.001
**HbA1C (%)**	1.65**	(1.35–2.02)	<0.001
**sVCAM-1(pg/ml)**	1.00**	(1.00–1.01)	<0.001
**CTRP3 (ng/ml)**	0.95**	(0.93–0.97)	<0.001
**CTRP5 (ng/ml)**	0.96**	(0.95–0.98)	<0.001

Values were determined by using univariate logistics regression analysis.

BMI, body mass index; SBP, systolic blood pressure; DBP, diastolic blood pressure; HbA1c, glycated hemoglobin A1c; HDL, high-density lipoprotein; LDL, low-density lipoprotein; TCH = total cholesterol; TG = triglycerides; CTRP, C1q/TNF-related protein.

**p<0.01.

**Table 3:** Correlation between DR and CTRPs.

Variable	Multivariate Analysis		
	OR	95%Cl	P
**Age (years)**	0.98	(0.92–1.05)	0.60
**Glucose (mmol/L)**	0.87	(0.71–1.08)	0.20
**HbA1C (%)**	0.91	(0.64–1.31)	0.63
**sVCAM-1(pg/ml)**	1.00	(1.00–1.01)	0.28
**CTRP3 (ng/ml)**	1.04*	(1.01–1.08)	0.01
**CTRP5 (ng/ml)**	1.02	(1.00–1.04)	0.08

Values were determined by using multivariate logistics regression analysis.

HbA1c, glycated hemoglobin A1c; sVCAM-1, soluble vascular cell adhesion molecule-1; CTRP, C1q/TNF-related protein.

*p<0.05.

### CTRP3 can serve as diagnostic biomarker for DR

**Fig 1:**
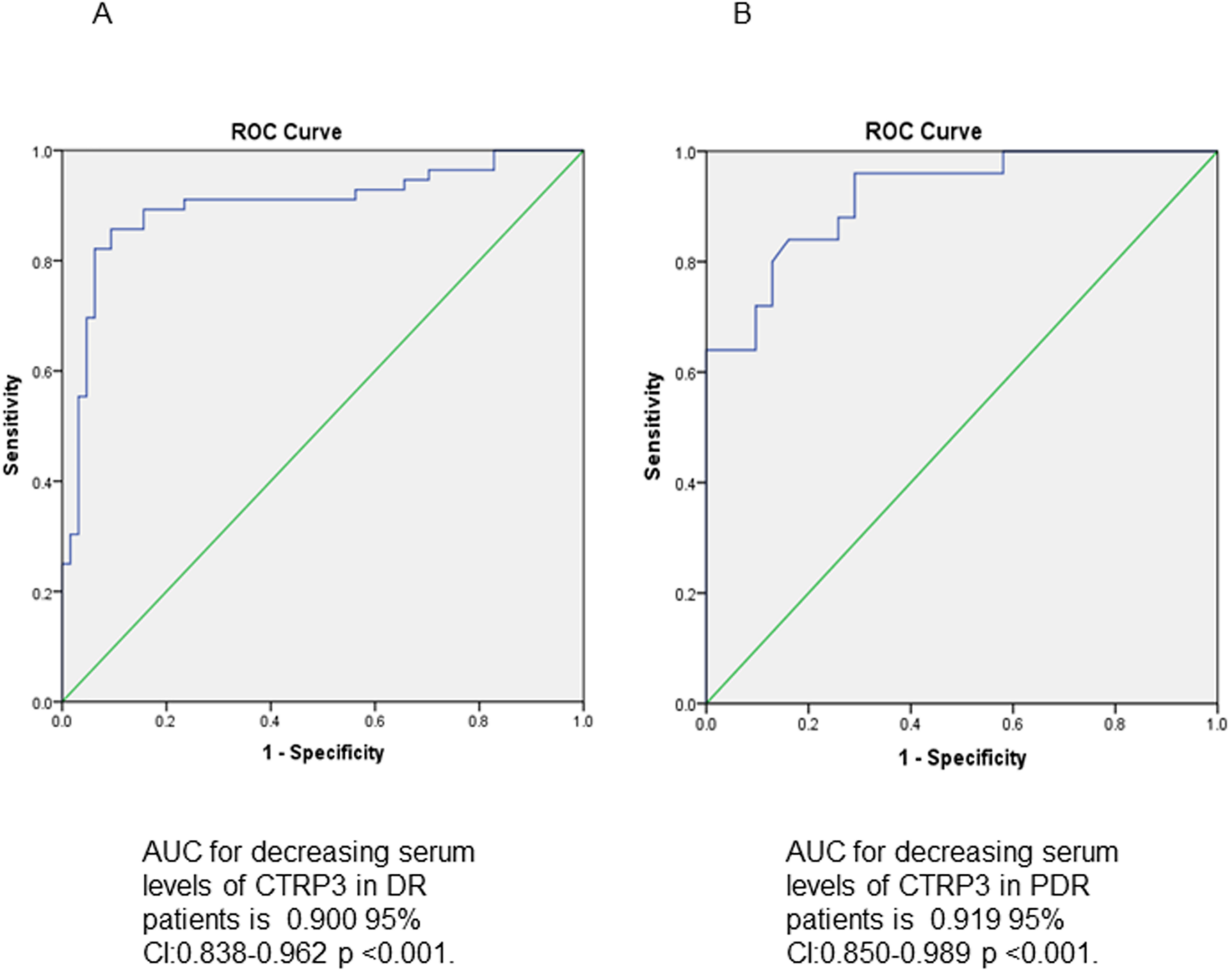
ROC curves, for DR/PDR diagnosis, by circulating CTRP3 level. (A) ROC curve analysis revealed that CTRP3 distinguishes patients with and without DR, AUC = 0.900 (95% CI 0.838–0.962, P<0.001). (B) CTRP3 distinguishes PDR from NPDR, AUC = 0.919 (95% CI 0.850–0.989, P<0.001).

A receiver operating characteristic (ROC) curve was constructed for evaluation of the diagnostic value of CTRP3 for DR. The AUC (are under the curve) was 0.900 (95% confidence interval 0.838–0.962, P<0.001, Fig 1A, n = 120), confirming excellent specificity and sensitivity of CTRP3 as diagnostic biomarker for DR.

Furthermore, in a ROC constructed for evaluation of the diagnostic value of CTRP3 for severity of DR, the AUC was 0.919 (95% CI 0.850~0.989, P<0.001 Fig 1B), suggesting CTRP3 may predict DR severity, distinguishing PDR from NPDR. Taken together, this data suggests serum CTRP3 level may be a marker of DR, and is associated with DR severity.

### sVCAM-1 is inversely associated with CTRP3

To determine the etiology of decreased CTRP3 in DR patients, and examine whether CTRP3 is associated with other risk factors, we analyzed the relationship between CTRP3 and other covariates. Spearman’s Rank correlation analysis revealed decreased serum CTRP3 was associated with glucose, HbA1C, sVCAM-1, and CTRP5 in all subjects (Table 4). Furthermore, in T2DM patients, serum CTRP3 is associated with glucose, sVCAM-1, and CTRP5. However, in DR patients, Serum CTRP3 is inversely associated with sVCAM-1 alone (Table 4 and Table D in S1 File), suggesting inflammation is strongly associated with decreased CTRP3.

**Table 4:** Correlation analysis of variables associated with circulating CTRP3.

Variables/Subjects	All (n = 120)			T2DM (n = 90)			DR (n = 56)		
	r	p		r	p		r		p
**Age (years)**	-0.014		0.883	-0.028		0.796	0.106	0.436	
**Gender**	0.028		0.765	-0.054		0.613	-0.090	0.507	
**BMI (kg/m2)**	-0.055		0.548	-0.047		0.658	0.120	0.378	
**Triglycerides (mmol/L)**	0.055		0.553	-0.041		0.702	-0.014	0.921	
**TCH (mmol/L)**	0.024		0.794	-0.165		0.120	-0.142	0.295	
**HDL-cholesterol (mmol/L)**	-0.084		0.360	0.016		0.882	-0.015	0.910	
**LDL-cholesterol (mmol/L)**	0.067		0.446	-0.058		0.586	0.003	0.982	
**TCH/HDL**	0.085		0.355	-0.086		0.423	-0.072	0.596	
**TG/HDL**	0.013		0.888	-0.116		0.276	-0.101	0.457	
**Glucose (mmol/L)**	-0.500		<0.001	-0.291		0.005	-0.035	0.796	
**HbA1C (%)**	-0.445		<0.001	-0.131		0.219	0.012	0.928	
**sVCAM-1(pg/ml)**	-0.707		<0.001	-0.663		<0.001	-0.597	<0.001	
**CTRP5(ng/ml)**	0.637		<0.001	0.520		<0.001	0.168	0.217	

Spearman’s Rank correlation analysis was used for analyzing associations between CTRP3 and variables.

BMI, body mass index; SBP, systolic blood pressure; DBP, diastolic blood pressure; HDL, high-density lipoprotein; LDL, low-density lipoprotein; TCH, total cholesterol; TG, triglycerides, HbA1c, glycated hemoglobin A1c.

### CTRP3 inhibited HGHL–induced VCAM-1 production in HRMECs

**Fig 2:**
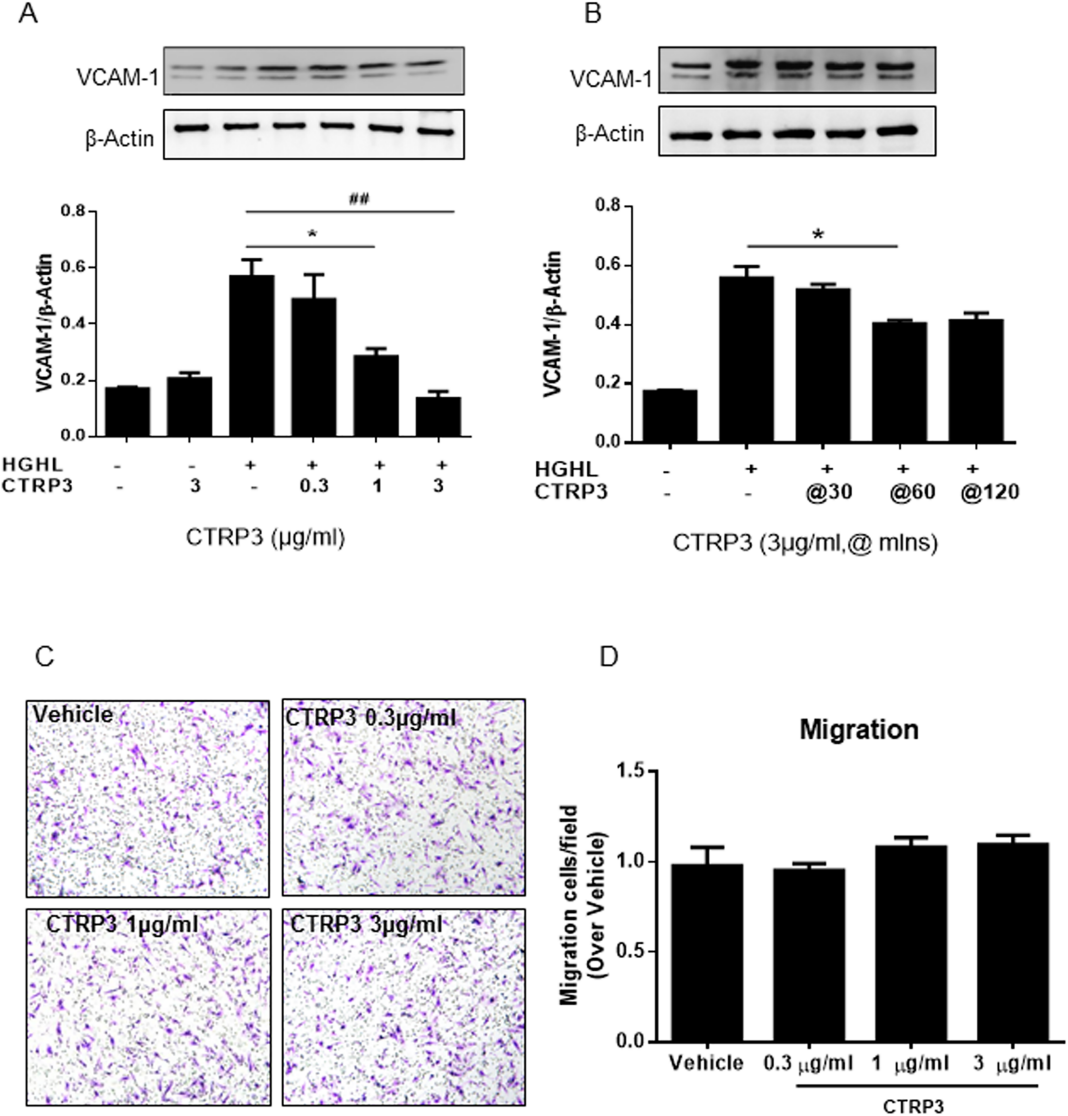
CTRP3 inhibits high glucose/high lipids (HGHL)-induced expression of VCAM-1 in a time- and dose-dependent manner. (A) HRMECs were subjected to high glucose/high lipid endothelial growth medium for 48 hours, incubated with CTRP3 (0.3, 1, 3μg/mL) for 60 minutes. VCAM-1 was inhibited in a concentration-dependent manner. (B) HRMECs were subjected to various periods of CTRP3 treatment (30, 60, and 120 minutes). VCAM-1 significantly decreased after 15 minutes, with decreasing trend for 120 total minutes. (C) HRMECs were placed in transwell migration chambers containing filters coated with matrigel. To stimulate migration, different concentrations of CTRP3 (0, 0.3, 1, 3μg/ml) were added to the lower chamber. Transwell chambers were stained with crystal violet and imaged after 8 hours. (D) The average number of migrated cells was quantified compared to vehicle control. Results are expressed as means±SD from 5–6 independent experiments. Each experiment was repeated 3 times. ^*^, P<0.05; ^##^, P<0.01 vs respective control.

Given the strong inverse association between CTRP3 and DR, we next determined the *in vitro* effects of CTRP3 upon adhesive molecules in human retinal microvascular endothelial cells (HRMECs) in the diabetic condition. First, we treated HRMECs with HGHL to mimic the diabetic condition. Next, HRMECs were subjected to CTRP3 treatment at different doses (0.3, 1, and 3μg/ml). CTRP3 inhibited VCAM-1 expression in a dose and time dependent manner (Fig 2A and 2B). However, CTRP3 administration did not change ICAM-1 (data not shown). To evaluate the effect of CTRP3 on cell migration, we performed a transwell migration assay, which is routinely employed for study of cellular migratory response to specific signal stimuli. HRMECs were placed in transwell migration chambers, and the population of cells migrating through the matrigel was ascertained. The population of cells migrating through the matrigel after CTRP3 treatment was not significantly altered compared to control (Fig 2C and 2D). Taken together, these results thus suggest CTRP3 1) is an inhibitor of VCAM-1, a protein represents for inflammatory processes involved in DR pathophysiology, and 2) has no effect upon HRMECs migration.

### Intact AMPK signaling necessary for CTRP3-mediated inhibition of HGHL-induced VCAM-1 expression

**Fig 3:**
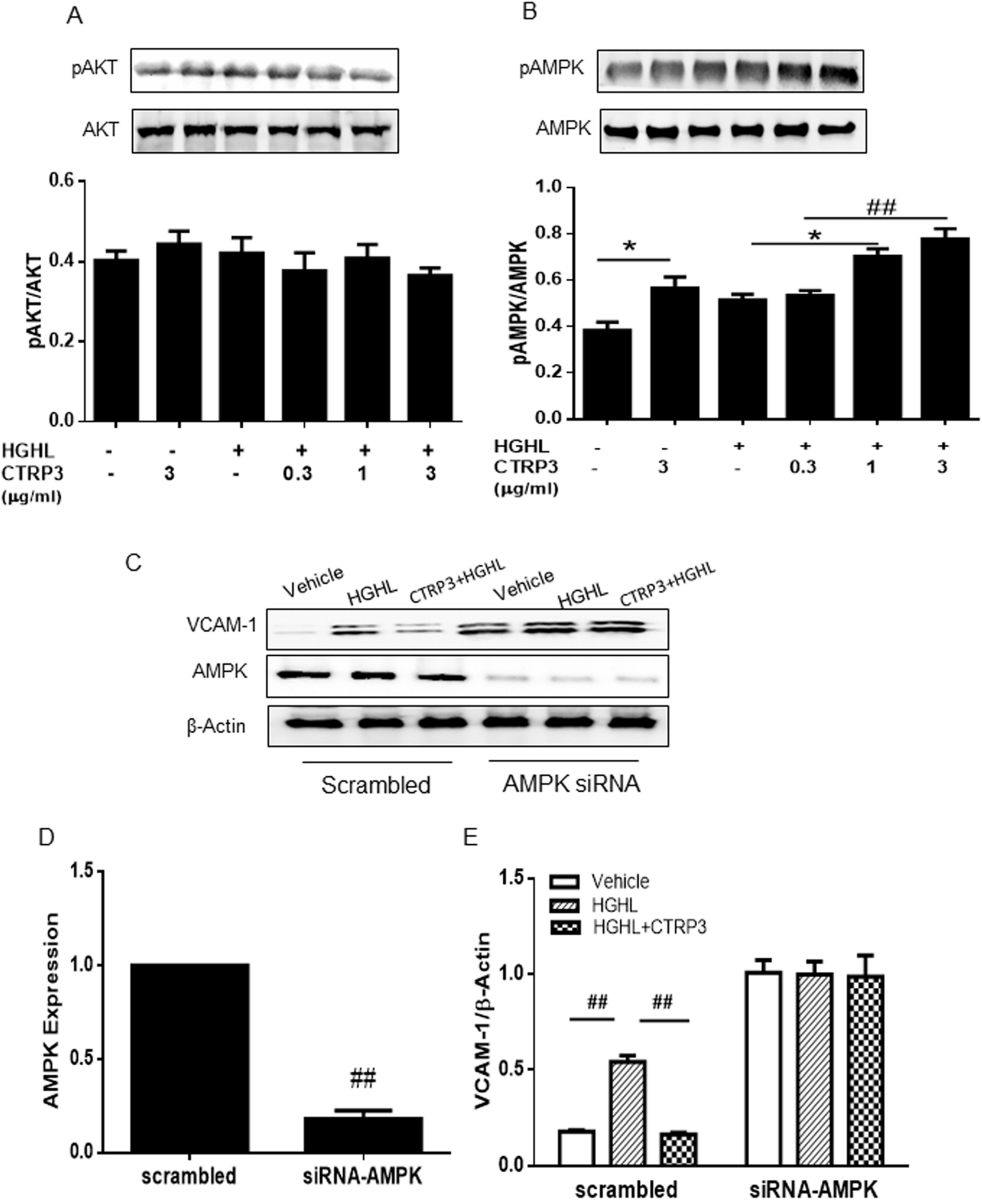
CTRP3 inhibited HGHL induced VCAM-1 production in an AMPK dependent manner. (A) HRMECs were incubated with HGHL for 48 hours, and subjected to CTRP3 administration (doses 0.3, 1, and 3μg/ml). (B) Akt phosphorylation was determined after 15 minutes. (C) AMPK phosphorylation was determined after 48 hours of HGHL incubation, followed by 15 minutes of CTRP3 treatment (doses 0.3, 1, and 3μg/ml). (D) Western blot analysis confirmed successful knockdown of AMPK by siRNA (>80% knockdown) E) AMPK knockdown blocked CTRP3 (3μg/ml, 1 hour treatment)-mediated inhibition of VCAM-1 expression induced by HGHL (48 hours treatment). Results are expressed as means±SD from 5–6 independent experiments. Each experiment was repeated 3 times. *p<0.05; ##, p<0.01 vs respective control.

AMPK regulates energy metabolism, with central importance in studies concerning diabetes and related metabolic diseases [26, 27]. We further detect AMPK’s role in CTRP3’s influence on VCAM-1 production. The results presented in Fig 3A and 3B showed that CTRP3 stimulates phosphorylation of AMPK, but not Akt. When we determined the effect of AMPK upon HGHL-induced VCAM-1 expression, we found that after successful AMPK knockdown by siRNA (>90% by Western analysis Fig 3D), CTRP3 (at highest dose 3μg/mL) does not inhibit VCAM-1 expression induced by HGHL in HRMECs (Fig 3C and 3E).

## Discussion

The present study demonstrated that CTRP3, but not CTRP5, has significant association with DR and its severity PDR. Additionally, CTRP3 inhibits HGHL-induced VCAM-1 expression in an AMPK-dependent manner in HRMECs, suggesting CTRP3 may have a role in the inflammatory processes of DR. This is the first study to investigate serum CTRPs and DR in the diabetic population.

Adipokines are associated with chronic proliferative vascular and inflammatory processes in diabetes. In recent years, many studies have investigated the relationship between CTRPs and diabetes [15]. CTRP3 promotes proliferation and migration of endothelial cells [28], but heretofore, no data concerning the role of CTRP3 in DR and PDR has been reported. In our study, we demonstrate CTRP3 may serve as a biomarker for DR, and also predict the severity of DR, i.e, PDR. Circulating CTRP3 and CTRP5 levels were significantly decreased in the T2DM (without DR) and DR patient groups compared to control. This association remained significant for DR and CTRP3 after adjustment for gender, BMI, HDL, LDL, and TG levels. Circulating CTRP3 levels were predictive of PDR (a condition of advanced DG) independently by ROC and AUC analysis, consistent with observations reported by Bo et al, who cite decreased plasma CTRP3 in newly diagnosed T2DM patients[15]. Adiponectin (a CTRP3 paralog) levels are markedly decreased in T2DM patients compared to control [29], and there is association between retinal blood flow and hypoadiponectinemia in DR patients [30].

Preclinical investigations demonstrate VCAM-1 levels may reflect endothelial cell damage [31]. Vitreous sVCAM-1 levels were significantly greater in PDR patients compared to control [32]. In consistence with those reports, our data supports sVCAM-1 levels is upregulated in DR patients. Furthermore, serum CTRP3 levels remain inversely correlated with sVCAM-1 levels after adjustment of other risk factors. Surprisingly, the correlation between sVCAM-1 and DR in our study was negative. Interference by other comorbidities in DR patients may explain this discrepancy.

We demonstrated CTRP3 inhibited VCAM-1 expression in a dose- and time-dependent manner, but did not significantly affect ICAM-1 in HRMECs, a finding that differs from the CTRP3 paralog adiponectin, which inhibits both ICAM-1 and VCAM-1 expression [33]. This reflects CTRP3 may serve as a more selective mediator in preventing inflammatory nature in the pathogenesis of diabetic microangiopathy. It has been reported the CTRP family may promote angiogenesis [34]. However, our study demonstrated that CTRP3 exerted no influence upon HRMEC migration, suggesting CTRP3 is not responsible for facilitating angiogenesis in HRMECs. Cell type and stimuli conditions may be responsible for the effect we observed. Therefore, our study provides evidence that CTRP3 has anti-inflammatory effect, but fails to promote migration, and future study is warranted. Adiponectin exerts its anti-inflammatory effect via AMPK-dependent signaling [24, 33] and CTRP family members have been demonstrated to activate phosphorylation via AMPK and Akt signaling [7, 35]. In the current study, we provide evidence that the ability of CTRP3, the analogue of adiponectin, to reduce VCAM-1 expression is to be mediated through its ability to activate AMPK pathway, in HGHL-treated HRMECs. Of note, we employed supra-physiologic concentrations of CTRP3 in our *in vitro* experiments, as done in the literature [36]. Recombinant CTRP3 may activate downstream signaling in greater doses than physiologic CTRP3. Additionally, we desired to identify the proper dose of CTRP3 carrying therapeutic anti-inflammatory potential, and therefore determined the response of HRMECs to CTRP3 in dose-dependent fashion.

Our study has several limitations. Firstly, the sample size was relatively small and was a Chinese population. Therefore, generalizing our results for the entire human population is not immediately possible. Secondly, our investigation was a case control study. It was not a large scale prospective randomized double blinded study of DR patients evaluative of a specific therapeutic intervention. Considerable work must still be completed before such a study can be conducted.

In conclusion, we have demonstrated that circulating CTRP3 may serve a valuable role as a biomarker screening for diabetic retinopathy, and may be an indicator of DR severity. Furthermore, CTRP3 inhibits HGHL-induced VCAM-1 expression in an AMPK-dependent manner in HRMECs, suggesting the novel therapeutic potential of CTRP3 for the treatment of DR, a serious and widespread complication of diabetes.

## Supporting information

S1 File Datasets for Patients’ characteristics analysis and experimental studies. **Table A.** General characteristics of study subjects. **Table B.** Correlation analysis between DR and laboratory characteristics. **Table C.** Correlation between DR and CTRPs. **Table D.** Correlation analysis of variables associated with circulating CTRP3. All, n = 120. **Table E.** Correlation analysis of variables associated with circulating CTRP3. T2DM, n = 90. **Table F.** Correlation analysis of variables associated with circulating CTRP3. DR, n = 56. **Fig A.** ROC curves, for DR/PDR diagnosis, by circulating CTRP3 level. (1) ROC curve for DR diagnosis by circulating CTRP3 level. (2) ROC curve for PDR diagnosis by circulating CTRP3. **Fig B.** CTRP3 inhibits high glucose/high lipids (HGHL)-induced expression of VCAM-1 in a time- and dose-dependent manner. (1) VCAM-1 was inhibited in a concentration-dependent manner following CTRP3 administration. (2) VCAM-1 significantly decreased after 15 minutes post CTRP3 treatment. **Fig C.** CTRP3 inhibited HGHL induced VCAM-1 production in an AMPK dependent manner. (1) Akt phosphorylation was determined after different dose CTRP3 administration. (2) AMPK phosphorylation was determined after 48 hours of HGHL incubation, followed by CTRP3 treatment. (3) AMPK knockdown blocked CTRP3-mediated inhibition of VCAM-1 expression induced by HGHL.
(PDF)
