# Supplementary material for: CTRP3 is a novel biomarker for diabetic retinopathy and inhibits HGHL-induced VCAM-1 expression in an AMPK-dependent manner
Source: PLoS One. 2017 Jun 20;12(6):e0178253. doi: 10.1371/journal.pone.0178253 (PMC5478095; doi:10.1371/journal.pone.0178253)

S1 File. Datasets for Patients' characteristics analysis and experimental studies.

Table A. General Characteristics of Study Subjects.

Descriptives

		N	Mean	Std. Deviation	Std. Error	95% Confidence Interval for Mean		Minimum	Maximum
						Lower Bound	Upper Bound		
Age	1.00	30	50.5333	18.58760	3.39362	43.5926	57.4741	17.00	78.00
	2.00	34	55.4706	9.05283	1.55255	52.3119	58.6293	36.00	70.00
	3.00	31	58.7097	11.23445	2.01777	54.5888	62.8305	31.00	75.00
	4.00	25	58.2400	11.56244	2.31249	53.4673	63.0127	29.00	75.00
	Total	120	55.6500	13.27261	1.21162	53.2509	58.0491	17.00	78.00
BMI	1.00	30	23.3667	2.96128	.54065	22.2609	24.4724	17.60	27.50
	2.00	34	22.7659	3.01005	.51622	21.7156	23.8161	19.30	30.40
	3.00	31	24.1642	3.98706	.71610	22.7017	25.6267	16.90	32.40
	4.00	25	23.9920	2.58456	.51691	22.9251	25.0589	20.30	28.70
	Total	120	23.5328	3.21365	.29336	22.9519	24.1136	16.90	32.40
TG	1.00	30	4.7287	.76915	.14043	4.4415	5.0159	3.49	6.10
	2.00	34	4.9429	1.21198	.20785	4.5201	5.3658	3.28	8.32
	3.00	31	4.6881	1.31165	.23558	4.2069	5.1692	2.90	8.72
	4.00	25	4.8768	1.12112	.22422	4.4140	5.3396	3.07	6.85
	Total	120	4.8098	1.11815	.10207	4.6076	5.0119	2.90	8.72
TCH	1.00	30	1.9760	.95567	.17448	1.6191	2.3329	.84	4.51
	2.00	34	1.5229	.81491	.13976	1.2386	1.8073	.13	3.25
	3.00	31	1.7187	1.39498	.25055	1.2070	2.2304	.54	8.26
	4.00	25	1.9488	.91180	.18236	1.5724	2.3252	.76	4.29
	Total	120	1.7755	1.04904	.09576	1.5859	1.9651	.13	8.26
HDL	1.00	30	1.0760	.22074	.04030	.9936	1.1584	.71	1.49
	2.00	34	1.3571	.73565	.12616	1.1004	1.6137	.72	3.97
	3.00	31	1.1161	.20402	.03664	1.0413	1.1910	.75	1.43
	4.00	25	1.1788	.29655	.05931	1.0564	1.3012	.63	1.86
	Total	120	1.1874	.45041	.04112	1.1060	1.2688	.63	3.97
LDL	1.00	30	2.8107	.85423	.15596	2.4917	3.1296	1.35	4.36
	2.00	34	2.6476	.69178	.11864	2.4063	2.8890	1.28	3.67
	3.00	31	2.7126	1.03199	.18535	2.3340	3.0911	.65	5.56
	4.00	25	2.8444	.98573	.19715	2.4375	3.2513	1.19	4.86
	Total	120	2.7462	.88382	.08068	2.5864	2.9059	.65	5.56
TGHDL	1.00	30	4.4653	1.07726	.19668	4.0631	4.8676	2.77	6.80
	2.00	34	4.0106	1.01592	.17423	3.6561	4.3651	2.09	5.87
	3.00	31	4.2519	1.08936	.19566	3.8524	4.6515	2.53	6.97
	4.00	25	4.3308	1.34630	.26926	3.7751	4.8865	1.65	6.43
	Total	120	4.2533	1.12391	.10260	4.0502	4.4565	1.65	6.97
TCHLDL	1.00	30	2.0567	1.55706	.28428	1.4752	2.6381	.32	6.42
	2.00	34	1.4418	.93797	.16086	1.1145	1.7690	.03	3.95
	3.00	31	1.5561	1.22196	.21947	1.1079	2.0043	.38	6.60
	4.00	25	1.7516	1.12976	.22595	1.2853	2.2179	.47	6.16
	Total	120	1.6896	1.23516	.11275	1.4663	1.9128	.03	6.60
HbA1C	1.00	30	4.8400	.73607	.13439	4.5651	5.1149	3.90	5.90
	2.00	34	8.0800	1.82028	.31218	7.4449	8.7151	5.16	11.17
	3.00	31	9.6016	2.30797	.41452	8.7550	10.4482	6.25	16.89
	4.00	25	8.3484	2.00755	.40151	7.5197	9.1771	4.48	12.00
	Total	120	7.7190	2.51791	.22985	7.2639	8.1741	3.90	16.89
Glucose	1.00	30	4.5533	.63286	.11554	4.3170	4.7896	3.90	6.10
	2.00	34	8.8159	2.75633	.47271	7.8542	9.7776	5.20	14.80
	3.00	31	13.9323	6.04987	1.08659	11.7131	16.1514	4.40	32.40
	4.00	25	12.5124	6.53420	1.30684	9.8152	15.2096	3.84	29.00
	Total	120	9.8421	5.76637	.52639	8.7998	10.8844	3.84	32.40
VCAM1	1.00	30	10055.4667	1130.68267	206.43347	9633.2628	10477.6705	7668.00	11573.00
	2.00	34	14789.0000	3486.56702	597.94131	13572.4793	16005.5207	10876.00	25001.00
	3.00	31	18362.4516	4686.16621	841.66030	16643.5520	20081.3513	1293.00	29080.00
	4.00	25	23835.5200	3061.85476	612.37095	22571.6485	25099.3915	19894.00	29319.00
	Total	120	16413.4500	5883.60867	537.09753	15349.9432	17476.9568	1293.00	29319.00
CTRP3	1.00	30	187.2467	51.91381	9.47812	167.8617	206.6316	81.21	289.00
	2.00	34	142.3118	13.80553	2.36763	137.4948	147.1287	123.60	170.30
	3.00	31	120.9226	30.68758	5.51165	109.6663	132.1789	83.80	199.30
	4.00	25	76.8308	18.20502	3.64100	69.3161	84.3455	50.00	119.30
	Total	120	134.3781	49.79363	4.54552	125.3775	143.3787	50.00	289.00
CTRP5	1.00	30	486.1320	88.13295	16.09080	453.2226	519.0414	370.62	685.03
	2.00	34	288.0394	20.63971	3.53968	280.8379	295.2409	245.56	332.24
	3.00	31	251.5235	52.89726	9.50063	232.1207	270.9264	164.89	407.45
	4.00	25	231.5872	39.46098	7.89220	215.2985	247.8759	109.49	296.07
	Total	120	316.3684	114.58922	10.46052	295.6555	337.0813	109.49	685.03

Hypothesis Test Summary

	Null Hypothesis	Test	Sig.	Decision
1	The distribution of Male is the same across categories of Group.	Independent-Samples Kruskal-Wallis Test	.650	Retain the null hypothesis.
2	The distribution of Hypertension is the same across categories of Group.	Independent-Samples Kruskal-Wallis Test	.340	Retain the null hypothesis.
3	The distribution of Diabeticnephropathy is the same across categories of Group.	Independent-Samples Kruskal-Wallis Test	.000	Reject the null hypothesis.
4	The distribution of Age is the same across categories of Group.	Independent-Samples Kruskal-Wallis Test	.187	Retain the null hypothesis.
5	The distribution of BMI is the same across categories of Group.	Independent-Samples Kruskal-Wallis Test	.224	Retain the null hypothesis.
6	The distribution of TG is the same across categories of Group.	Independent-Samples Kruskal-Wallis Test	.689	Retain the null hypothesis.
7	The distribution of TCH is the same across categories of Group.	Independent-Samples Kruskal-Wallis Test	.077	Retain the null hypothesis.
8	The distribution of HDL is the same across categories of Group.	Independent-Samples Kruskal-Wallis Test	.260	Retain the null hypothesis.
9	The distribution of LDL is the same across categories of Group.	Independent-Samples Kruskal-Wallis Test	.892	Retain the null hypothesis.
10	The distribution of TGHDL is the same across categories of Group.	Independent-Samples Kruskal-Wallis Test	.543	Retain the null hypothesis.
11	The distribution of TCHLDL is the same across categories of Group.	Independent-Samples Kruskal-Wallis Test	.148	Retain the null hypothesis.

Asymptotic significances are displayed. The significance level is .05.

Table B. Correlation between DR and laboratory characteristics

Variables in the Equation

		B	S.E.	Wald	df	Sig.	Exp(B)	95% C.I.for EXP(B)	
								Lower	Upper
Step 1[a]	Age	.032	.015	4.683	1	.030	1.033	1.003	1.063
	Constant	-1.937	.860	5.077	1	.024	.144		

a. Variable(s) entered on step 1: Age.

Variables in the Equation

		B	S.E.	Wald	df	Sig.	Exp(B)	95% C.I.for EXP(B)	
								Lower	Upper
Step 1[a]	Male	-.244	.368	.438	1	.508	.784	.381	1.613
	Constant	.000	.272	.000	1	1.000	1.000		

a. Variable(s) entered on step 1: Male.

Variables in the Equation

		B	S.E.	Wald	df	Sig.	Exp(B)	95% C.I.for EXP(B)	
								Lower	Upper
Step 1[a]	Hypertension	.376	.458	.674	1	.412	1.457	.593	3.576
	Constant	-.209	.205	1.038	1	.308	.811		

a. Variable(s) entered on step 1: Hypertension.

Variables in the Equation

		B	S.E.	Wald	df	Sig.	Exp(B)	95% C.I.for EXP(B)	
								Lower	Upper
Step 1[a]	BMI	.103	.059	3.086	1	.079	1.109	.988	1.245
	Constant	-2.572	1.401	3.367	1	.067	.076		

a. Variable(s) entered on step 1: BMI.

Variables in the Equation

		B	S.E.	Wald	df	Sig.	Exp(B)	95% C.I.for EXP(B)	
								Lower	Upper
Step 1[a]	TG	-.057	.165	.118	1	.731	.945	.684	1.306
	Constant	.140	.814	.029	1	.864	1.150		

a. Variable(s) entered on step 1: TG.

Variables in the Equation

		B	S.E.	Wald	df	Sig.	Exp(B)	95% C.I.for EXP(B)	
								Lower	Upper
Step 1[a]	TCH	.079	.176	.201	1	.654	1.082	.766	1.528
	Constant	-.274	.362	.571	1	.450	.760		

a. Variable(s) entered on step 1: TCH.

Variables in the Equation

		B	S.E.	Wald	df	Sig.	Exp(B)	95% C.I.for EXP(B)	
								Lower	Upper
Step 1[a]	HDL	-.446	.466	.914	1	.339	.640	.257	1.597
	Constant	.392	.575	.465	1	.495	1.480		

a. Variable(s) entered on step 1: HDL.

Variables in the Equation

		B	S.E.	Wald	df	Sig.	Exp(B)	95% C.I.for EXP(B)	
								Lower	Upper
Step 1[a]	LDL	.061	.208	.086	1	.769	1.063	.707	1.598
	Constant	-.302	.600	.252	1	.615	.740		

a. Variable(s) entered on step 1: LDL.

Variables in the Equation

		B	S.E.	Wald	df	Sig.	Exp(B)	95% C.I.for EXP(B)	
								Lower	Upper
Step 1[a]	TGHDL	.051	.164	.096	1	.757	1.052	.763	1.449
	Constant	-.349	.720	.235	1	.628	.705		

a. Variable(s) entered on step 1: TGHDL.

Variables in the Equation

		B	S.E.	Wald	df	Sig.	Exp(B)	95% C.I.for EXP(B)	
								Lower	Upper
Step 1[a]	TCHLDL	-.058	.150	.148	1	.701	.944	.703	1.267
	Constant	-.036	.312	.013	1	.908	.964		

a. Variable(s) entered on step 1: TCHLDL.

Variables in the Equation

		B	S.E.	Wald	df	Sig.	Exp(B)	95% C.I.for EXP(B)	
								Lower	Upper
Step 1[a]	HbA1C	.501	.102	24.155	1	.000	1.650	1.351	2.015
	Constant	-4.009	.817	24.082	1	.000	.018		

a. Variable(s) entered on step 1: HbA1C.

Variables in the Equation

		B	S.E.	Wald	df	Sig.	Exp(B)	95% C.I.for EXP(B)	
								Lower	Upper
Step 1[a]	Glucose	.393	.077	26.140	1	.000	1.481	1.274	1.722
	Constant	-3.738	.708	27.880	1	.000	.024		

a. Variable(s) entered on step 1: Glucose.

Variables in the Equation

		B	S.E.	Wald	df	Sig.	Exp(B)	95% C.I.for EXP(B)	
								Lower	Upper
Step 1[a]	Glucose	.393	.077	26.140	1	.000	1.481	1.274	1.722
	Constant	-3.738	.708	27.880	1	.000	.024		

a. Variable(s) entered on step 1: Glucose.

Variables in the Equation

		B	S.E.	Wald	df	Sig.	Exp(B)	95% C.I.for EXP(B)	
								Lower	Upper
Step 1[a]	CTRP3	-.050	.009	28.240	1	.000	.952	.934	.969
	Constant	6.234	1.196	27.148	1	.000	509.667		

a. Variable(s) entered on step 1: CTRP3.

Variables in the Equation

		B	S.E.	Wald	df	Sig.	Exp(B)	95% C.I.for EXP(B)	
								Lower	Upper
Step 1[a]	CTRP5	-.036	.008	19.749	1	.000	.965	.950	.980
	Constant	10.022	2.206	20.640	1	.000	22527.662		

a. Variable(s) entered on step 1: CTRP5.

Table C. Correlation between DR and CTRPs

Variables in the Equation

		B	S.E.	Wald	df	Sig.	Exp(B)	95% C.I.for EXP(B)	
								Lower	Upper
Step 1[a]	Age	.018	.033	.277	1	.599	1.018	.953	1.086
	HbA1C	.090	.185	.236	1	.627	1.094	.761	1.573
	Glucose	.137	.106	1.645	1	.200	1.146	.930	1.412
	VCAM1	.000	.000	1.162	1	.281	1.000	1.000	1.000
	CTRP3	-.043	.016	7.782	1	.005	.958	.929	.987
	CTRP5	-.016	.009	3.093	1	.079	.984	.966	1.002
	Constant	5.209	3.956	1.734	1	.188	182.829		

a. Variable(s) entered on step 1: Age, HbA1C, Glucose, VCAM1, CTRP3, CTRP5.

Table D. Correlation analysis of variables associated with circulating CTRP3 All，n=120

Correlations

			Male	Age	BMI	TG	TCH	HDL	LDL	TGHDL	TCHLDL	HbA1C	Glucose	VCAM1	CTRP3	CTRP5	Hypertension
Spearman's rho	Male	Correlation Coefficient	1.000	.135	.074	.029	.114	-.223*	.060	.172	.075	-.077	-.020	.014	.028	.001	.034
		Sig. (2-tailed)	.	.143	.423	.757	.216	.015	.515	.061	.416	.402	.825	.879	.765	.987	.716
		N	120	120	120	120	120	120	120	120	120	120	120	120	120	120	120
	Age	Correlation Coefficient	.135	1.000	.111	.277**	.058	.061	.229*	.050	-.196*	.151	.246**	.205*	-.014	-.237**	.012
		Sig. (2-tailed)	.143	.	.227	.002	.527	.510	.012	.586	.032	.099	.007	.025	.883	.009	.896
		N	120	120	120	120	120	120	120	120	120	120	120	120	120	120	120
	BMI	Correlation Coefficient	.074	.111	1.000	.103	.212*	-.158	.116	.249**	.173	-.027	.049	.002	.026	.049	-.142
		Sig. (2-tailed)	.423	.227	.	.263	.020	.085	.207	.006	.059	.767	.593	.981	.779	.595	.123
		N	120	120	120	120	120	120	120	120	120	120	120	120	120	120	120
	TG	Correlation Coefficient	.029	.277**	.103	1.000	.241**	.330**	.827**	.447**	.005	-.124	-.079	-.090	.055	.084	.015
		Sig. (2-tailed)	.757	.002	.263	.	.008	.000	.000	.000	.961	.179	.393	.328	.553	.361	.870
		N	120	120	120	120	120	120	120	120	120	120	120	120	120	120	120
	TCH	Correlation Coefficient	.114	.058	.212*	.241**	1.000	-.412**	.127	.610**	.736**	-.149	-.093	-.152	.024	.137	-.065
		Sig. (2-tailed)	.216	.527	.020	.008	.	.000	.167	.000	.000	.104	.314	.097	.794	.137	.479
		N	120	120	120	120	120	120	120	120	120	120	120	120	120	120	120
	HDL	Correlation Coefficient	-.223*	.061	-.158	.330**	-.412**	1.000	.200*	-.607**	-.519**	.030	.083	.051	-.084	-.113	.043
		Sig. (2-tailed)	.015	.510	.085	.000	.000	.	.029	.000	.000	.742	.366	.577	.360	.221	.641
		N	120	120	120	120	120	120	120	120	120	120	120	120	120	120	120
	LDL	Correlation Coefficient	.060	.229*	.116	.827**	.127	.200*	1.000	.493**	.026	-.098	-.081	-.037	.067	.025	-.111
		Sig. (2-tailed)	.515	.012	.207	.000	.167	.029	.	.000	.776	.286	.382	.686	.466	.786	.229
		N	120	120	120	120	120	120	120	120	120	120	120	120	120	120	120
	TGHDL	Correlation Coefficient	.172	.050	.249**	.447**	.610**	-.607**	.493**	1.000	.527**	-.054	-.103	-.090	.013	.125	-.070
		Sig. (2-tailed)	.061	.586	.006	.000	.000	.000	.000	.	.000	.555	.262	.327	.888	.175	.449
		N	120	120	120	120	120	120	120	120	120	120	120	120	120	120	120
	TCHLDL	Correlation Coefficient	.075	-.196*	.173	.005	.736**	-.519**	.026	.527**	1.000	-.138	-.125	-.160	.007	.173	-.168
		Sig. (2-tailed)	.416	.032	.059	.961	.000	.000	.776	.000	.	.134	.172	.081	.936	.059	.066
		N	120	120	120	120	120	120	120	120	120	120	120	120	120	120	120
	HbA1C	Correlation Coefficient	-.077	.151	-.027	-.124	-.149	.030	-.098	-.054	-.138	1.000	.750**	.662**	-.445**	-.548**	.058
		Sig. (2-tailed)	.402	.099	.767	.179	.104	.742	.286	.555	.134	.	.000	.000	.000	.000	.531
		N	120	120	120	120	120	120	120	120	120	120	120	120	120	120	120
	Glucose	Correlation Coefficient	-.020	.246**	.049	-.079	-.093	.083	-.081	-.103	-.125	.750**	1.000	.717**	-.500**	-.676**	-.017
		Sig. (2-tailed)	.825	.007	.593	.393	.314	.366	.382	.262	.172	.000	.	.000	.000	.000	.850
		N	120	120	120	120	120	120	120	120	120	120	120	120	120	120	120
	VCAM1	Correlation Coefficient	.014	.205*	.002	-.090	-.152	.051	-.037	-.090	-.160	.662**	.717**	1.000	-.707**	-.764**	.191*
		Sig. (2-tailed)	.879	.025	.981	.328	.097	.577	.686	.327	.081	.000	.000	.	.000	.000	.037
		N	120	120	120	120	120	120	120	120	120	120	120	120	120	120	120
	CTRP3	Correlation Coefficient	.028	-.014	.026	.055	.024	-.084	.067	.013	.007	-.445**	-.500**	-.707**	1.000	.637**	-.013
		Sig. (2-tailed)	.765	.883	.779	.553	.794	.360	.466	.888	.936	.000	.000	.000	.	.000	.889
		N	120	120	120	120	120	120	120	120	120	120	120	120	120	120	120
	CTRP5	Correlation Coefficient	.001	-.237**	.049	.084	.137	-.113	.025	.125	.173	-.548**	-.676**	-.764**	.637**	1.000	-.057
		Sig. (2-tailed)	.987	.009	.595	.361	.137	.221	.786	.175	.059	.000	.000	.000	.000	.	.537
		N	120	120	120	120	120	120	120	120	120	120	120	120	120	120	120
	Hypertension	Correlation Coefficient	.034	.012	-.142	.015	-.065	.043	-.111	-.070	-.168	.058	-.017	.191*	-.013	-.057	1.000
		Sig. (2-tailed)	.716	.896	.123	.870	.479	.641	.229	.449	.066	.531	.850	.037	.889	.537	.
		N	120	120	120	120	120	120	120	120	120	120	120	120	120	120	120

*. Correlation is significant at the 0.05 level (2-tailed).

**. Correlation is significant at the 0.01 level (2-tailed).

Table E. Correlation analysis of variables associated with circulating CTRP3 T2DM，n=90

Correlations

			Male	Age	BMI	TG	TCH	HDL	LDL	TGHDL	TCHLDL	HbA1C	Glucose	VCAM1	CTRP3	CTRP5	Hypertension
Spearman's rho	Male	Correlation Coefficient	1.000	-.100	-.009	-.134	-.059	-.160	-.056	.015	.057	-.046	.037	.068	-.054	-.037	.071
		Sig. (2-tailed)	.	.351	.933	.209	.580	.131	.602	.892	.591	.668	.730	.526	.613	.730	.503
		N	90	90	90	90	90	90	90	90	90	90	90	90	90	90	90
	Age	Correlation Coefficient	-.100	1.000	.044	.098	-.034	.095	.067	-.085	-.096	.057	.309**	.174	-.028	-.155	-.032
		Sig. (2-tailed)	.351	.	.678	.357	.752	.374	.533	.428	.367	.594	.003	.102	.796	.145	.765
		N	90	90	90	90	90	90	90	90	90	90	90	90	90	90	90
	BMI	Correlation Coefficient	-.009	.044	1.000	.058	.186	-.131	.060	.221*	.170	-.091	.071	-.055	-.047	.104	-.139
		Sig. (2-tailed)	.933	.678	.	.588	.080	.218	.574	.036	.110	.394	.504	.606	.658	.328	.190
		N	90	90	90	90	90	90	90	90	90	90	90	90	90	90	90
	TG	Correlation Coefficient	-.134	.098	.058	1.000	.214*	.383**	.806**	.450**	.062	-.188	-.080	-.126	-.041	.147	.029
		Sig. (2-tailed)	.209	.357	.588	.	.043	.000	.000	.000	.559	.076	.453	.237	.702	.167	.787
		N	90	90	90	90	90	90	90	90	90	90	90	90	90	90	90
	TCH	Correlation Coefficient	-.059	-.034	.186	.214*	1.000	-.333**	.175	.580**	.902**	-.060	.028	-.056	-.165	.063	-.124
		Sig. (2-tailed)	.580	.752	.080	.043	.	.001	.098	.000	.000	.577	.792	.600	.120	.556	.244
		N	90	90	90	90	90	90	90	90	90	90	90	90	90	90	90
	HDL	Correlation Coefficient	-.160	.095	-.131	.383**	-.333**	1.000	.151	-.565**	-.605**	-.107	-.055	-.068	.016	-.008	.148
		Sig. (2-tailed)	.131	.374	.218	.000	.001	.	.157	.000	.000	.317	.605	.527	.882	.943	.165
		N	90	90	90	90	90	90	90	90	90	90	90	90	90	90	90
	LDL	Correlation Coefficient	-.056	.067	.060	.806**	.175	.151	1.000	.587**	.099	-.147	-.057	-.011	-.058	.000	-.099
		Sig. (2-tailed)	.602	.533	.574	.000	.098	.157	.	.000	.351	.167	.595	.917	.586	.998	.354
		N	90	90	90	90	90	90	90	90	90	90	90	90	90	90	90
	TGHDL	Correlation Coefficient	.015	-.085	.221*	.450**	.580**	-.565**	.587**	1.000	.670**	.010	-.004	-.039	-.116	.109	-.168
		Sig. (2-tailed)	.892	.428	.036	.000	.000	.000	.000	.	.000	.929	.970	.714	.276	.306	.114
		N	90	90	90	90	90	90	90	90	90	90	90	90	90	90	90
	TCHLDL	Correlation Coefficient	.057	-.096	.170	.062	.902**	-.605**	.099	.670**	1.000	-.037	-.010	-.055	-.086	.059	-.104
		Sig. (2-tailed)	.591	.367	.110	.559	.000	.000	.351	.000	.	.730	.924	.606	.423	.579	.330
		N	90	90	90	90	90	90	90	90	90	90	90	90	90	90	90
	HbA1C	Correlation Coefficient	-.046	.057	-.091	-.188	-.060	-.107	-.147	.010	-.037	1.000	.519**	.328**	-.131	-.045	-.025
		Sig. (2-tailed)	.668	.594	.394	.076	.577	.317	.167	.929	.730	.	.000	.002	.219	.674	.817
		N	90	90	90	90	90	90	90	90	90	90	90	90	90	90	90
	Glucose	Correlation Coefficient	.037	.309**	.071	-.080	.028	-.055	-.057	-.004	-.010	.519**	1.000	.474**	-.291**	-.357**	-.130
		Sig. (2-tailed)	.730	.003	.504	.453	.792	.605	.595	.970	.924	.000	.	.000	.005	.001	.223
		N	90	90	90	90	90	90	90	90	90	90	90	90	90	90	90
	VCAM1	Correlation Coefficient	.068	.174	-.055	-.126	-.056	-.068	-.011	-.039	-.055	.328**	.474**	1.000	-.663**	-.500**	.203
		Sig. (2-tailed)	.526	.102	.606	.237	.600	.527	.917	.714	.606	.002	.000	.	.000	.000	.055
		N	90	90	90	90	90	90	90	90	90	90	90	90	90	90	90
	CTRP3	Correlation Coefficient	-.054	-.028	-.047	-.041	-.165	.016	-.058	-.116	-.086	-.131	-.291**	-.663**	1.000	.520**	.020
		Sig. (2-tailed)	.613	.796	.658	.702	.120	.882	.586	.276	.423	.219	.005	.000	.	.000	.851
		N	90	90	90	90	90	90	90	90	90	90	90	90	90	90	90
	CTRP5	Correlation Coefficient	-.037	-.155	.104	.147	.063	-.008	.000	.109	.059	-.045	-.357**	-.500**	.520**	1.000	.076
		Sig. (2-tailed)	.730	.145	.328	.167	.556	.943	.998	.306	.579	.674	.001	.000	.000	.	.479
		N	90	90	90	90	90	90	90	90	90	90	90	90	90	90	90
	Hypertension	Correlation Coefficient	.071	-.032	-.139	.029	-.124	.148	-.099	-.168	-.104	-.025	-.130	.203	.020	.076	1.000
		Sig. (2-tailed)	.503	.765	.190	.787	.244	.165	.354	.114	.330	.817	.223	.055	.851	.479	.
		N	90	90	90	90	90	90	90	90	90	90	90	90	90	90	90

**. Correlation is significant at the 0.01 level (2-tailed).

*. Correlation is significant at the 0.05 level (2-tailed).

Table F. Correlation analysis of variables associated with circulating CTRP3 DR，n=56

Correlations

			Male	Age	BMI	TG	TCH	HDL	LDL	TGHDL	TCHLDL	HbA1C	Glucose	VCAM1	CTRP3	CTRP5	Hypertension
Spearman's rho	Male	Correlation Coefficient	1.000	-.250	.053	-.105	-.076	-.104	-.044	-.001	.027	-.126	.043	.093	-.045	-.004	.023
		Sig. (2-tailed)	.	.063	.698	.441	.576	.446	.746	.994	.846	.355	.752	.496	.740	.974	.868
		N	56	56	56	56	56	56	56	56	56	56	56	56	56	56	56
	Age	Correlation Coefficient	-.250	1.000	.058	.090	.057	.048	.160	-.019	-.013	.018	.306*	-.016	.106	-.126	-.193
		Sig. (2-tailed)	.063	.	.672	.507	.674	.725	.238	.892	.924	.893	.022	.905	.436	.356	.155
		N	56	56	56	56	56	56	56	56	56	56	56	56	56	56	56
	BMI	Correlation Coefficient	.053	.058	1.000	.055	.255	-.131	.020	.227	.228	-.213	-.027	-.232	.120	.219	-.196
		Sig. (2-tailed)	.698	.672	.	.689	.058	.336	.884	.093	.091	.116	.841	.085	.378	.105	.147
		N	56	56	56	56	56	56	56	56	56	56	56	56	56	56	56
	TG	Correlation Coefficient	-.105	.090	.055	1.000	.437**	.241	.941**	.668**	.330*	-.078	-.057	-.117	-.014	-.040	-.082
		Sig. (2-tailed)	.441	.507	.689	.	.001	.073	.000	.000	.013	.566	.677	.392	.921	.772	.546
		N	56	56	56	56	56	56	56	56	56	56	56	56	56	56	56
	TCH	Correlation Coefficient	-.076	.057	.255	.437**	1.000	-.241	.278*	.555**	.907**	-.147	.071	-.009	-.142	.237	-.064
		Sig. (2-tailed)	.576	.674	.058	.001	.	.073	.038	.000	.000	.281	.602	.947	.295	.078	.639
		N	56	56	56	56	56	56	56	56	56	56	56	56	56	56	56
	HDL	Correlation Coefficient	-.104	.048	-.131	.241	-.241	1.000	.197	-.499**	-.492**	.072	-.127	-.035	-.015	-.266*	.118
		Sig. (2-tailed)	.446	.725	.336	.073	.073	.	.147	.000	.000	.597	.351	.795	.910	.048	.387
		N	56	56	56	56	56	56	56	56	56	56	56	56	56	56	56
	LDL	Correlation Coefficient	-.044	.160	.020	.941**	.278*	.197	1.000	.641**	.210	-.104	-.067	-.158	.003	-.099	-.157
		Sig. (2-tailed)	.746	.238	.884	.000	.038	.147	.	.000	.121	.447	.625	.244	.982	.469	.248
		N	56	56	56	56	56	56	56	56	56	56	56	56	56	56	56
	TGHDL	Correlation Coefficient	-.001	-.019	.227	.668**	.555**	-.499**	.641**	1.000	.644**	-.056	.077	-.081	-.072	.221	-.217
		Sig. (2-tailed)	.994	.892	.093	.000	.000	.000	.000	.	.000	.682	.571	.554	.596	.102	.108
		N	56	56	56	56	56	56	56	56	56	56	56	56	56	56	56
	TCHLDL	Correlation Coefficient	.027	-.013	.228	.330*	.907**	-.492**	.210	.644**	1.000	-.186	.080	.002	-.101	.235	-.038
		Sig. (2-tailed)	.846	.924	.091	.013	.000	.000	.121	.000	.	.170	.559	.987	.457	.082	.781
		N	56	56	56	56	56	56	56	56	56	56	56	56	56	56	56
	HbA1C	Correlation Coefficient	-.126	.018	-.213	-.078	-.147	.072	-.104	-.056	-.186	1.000	.484**	.094	.012	.141	-.034
		Sig. (2-tailed)	.355	.893	.116	.566	.281	.597	.447	.682	.170	.	.000	.491	.928	.300	.803
		N	56	56	56	56	56	56	56	56	56	56	56	56	56	56	56
	Glucose	Correlation Coefficient	.043	.306*	-.027	-.057	.071	-.127	-.067	.077	.080	.484**	1.000	.235	-.035	-.172	-.098
		Sig. (2-tailed)	.752	.022	.841	.677	.602	.351	.625	.571	.559	.000	.	.081	.796	.205	.472
		N	56	56	56	56	56	56	56	56	56	56	56	56	56	56	56
	VCAM1	Correlation Coefficient	.093	-.016	-.232	-.117	-.009	-.035	-.158	-.081	.002	.094	.235	1.000	-.597**	-.207	.342**
		Sig. (2-tailed)	.496	.905	.085	.392	.947	.795	.244	.554	.987	.491	.081	.	.000	.126	.010
		N	56	56	56	56	56	56	56	56	56	56	56	56	56	56	56
	CTRP3	Correlation Coefficient	-.045	.106	.120	-.014	-.142	-.015	.003	-.072	-.101	.012	-.035	-.597**	1.000	.168	.054
		Sig. (2-tailed)	.740	.436	.378	.921	.295	.910	.982	.596	.457	.928	.796	.000	.	.217	.695
		N	56	56	56	56	56	56	56	56	56	56	56	56	56	56	56
	CTRP5	Correlation Coefficient	-.004	-.126	.219	-.040	.237	-.266*	-.099	.221	.235	.141	-.172	-.207	.168	1.000	.085
		Sig. (2-tailed)	.974	.356	.105	.772	.078	.048	.469	.102	.082	.300	.205	.126	.217	.	.533
		N	56	56	56	56	56	56	56	56	56	56	56	56	56	56	56
	Hypertension	Correlation Coefficient	.023	-.193	-.196	-.082	-.064	.118	-.157	-.217	-.038	-.034	-.098	.342**	.054	.085	1.000
		Sig. (2-tailed)	.868	.155	.147	.546	.639	.387	.248	.108	.781	.803	.472	.010	.695	.533	.
		N	56	56	56	56	56	56	56	56	56	56	56	56	56	56	56

*. Correlation is significant at the 0.05 level (2-tailed).

**. Correlation is significant at the 0.01 level (2-tailed).

Fig A. ROC curves, for DR/PDR diagnosis, by circulating CTRP3 level.

1

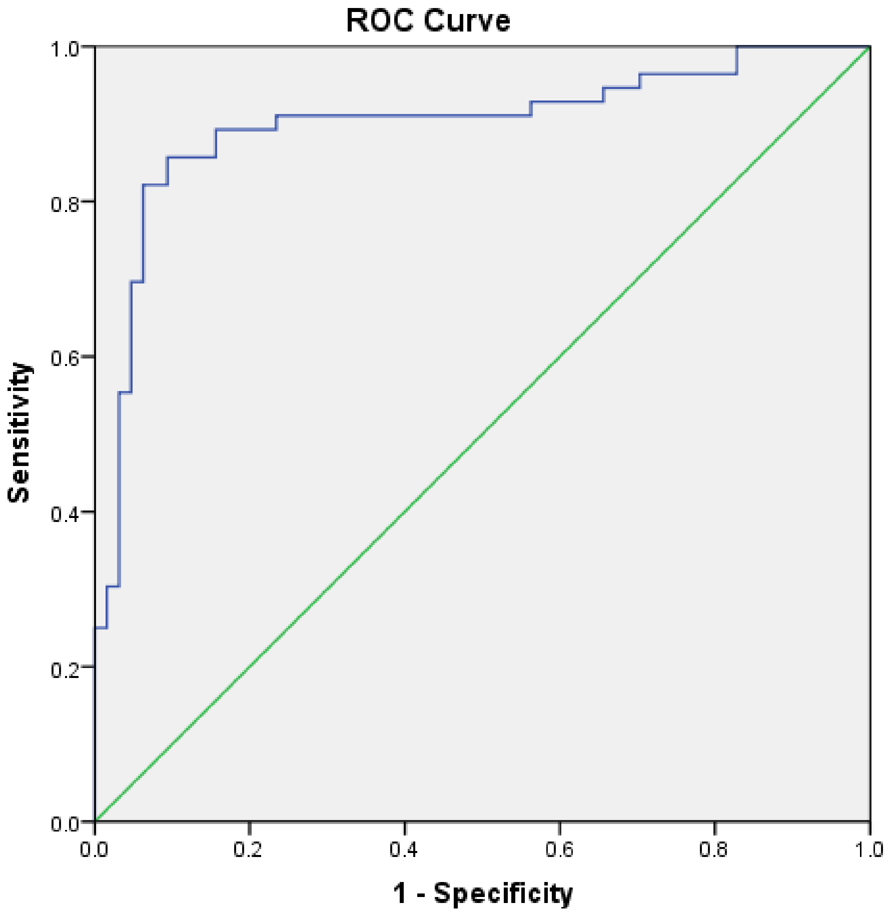

Area Under the Curve

Test Result Variable(s): CTRP3

Area	Std. Error[a]	Asymptotic Sig.[b]	Asymptotic 95% Confidence Interval	
			Lower Bound	Upper Bound
.900	.032	.000	.838	.962

a. Under the nonparametric assumption

b. Null hypothesis: true area = 0.5

Fig A. 2

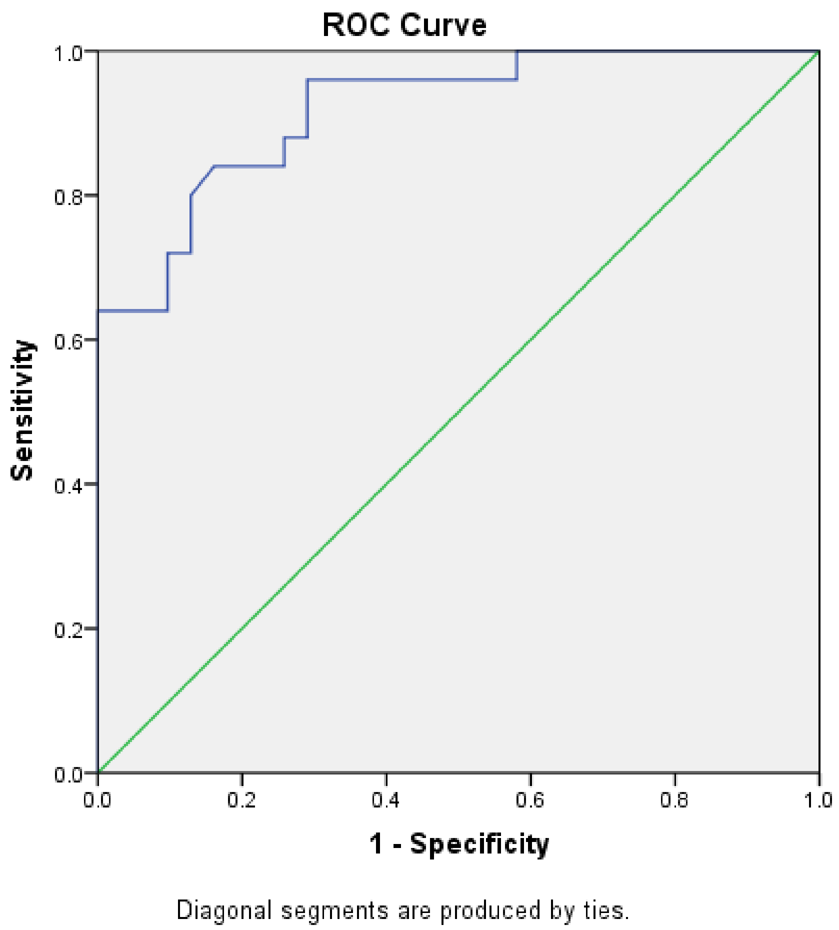

Area Under the Curve

Test Result Variable(s): CTRP3

Area	Std. Error[a]	Asymptotic Sig.[b]	Asymptotic 95% Confidence Interval	
			Lower Bound	Upper Bound
.919	.035	.000	.850	.989

The test result variable(s): CTRP3 has at least one tie between the positive actual state group and the negative actual state group. Statistics may be biased.

a. Under the nonparametric assumption

b. Null hypothesis: true area = 0.5

Fig B. CTRP3 inhibits high glucose/high lipids (HGHL)-induced expression of VCAM-1 in a time- and dose-dependent manner.

1

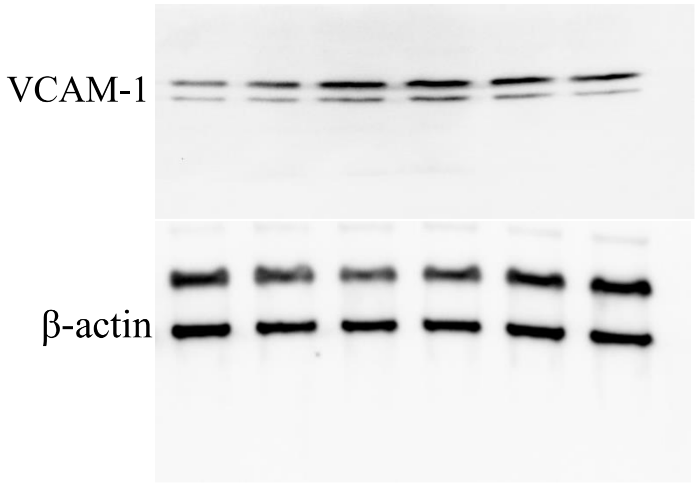

2

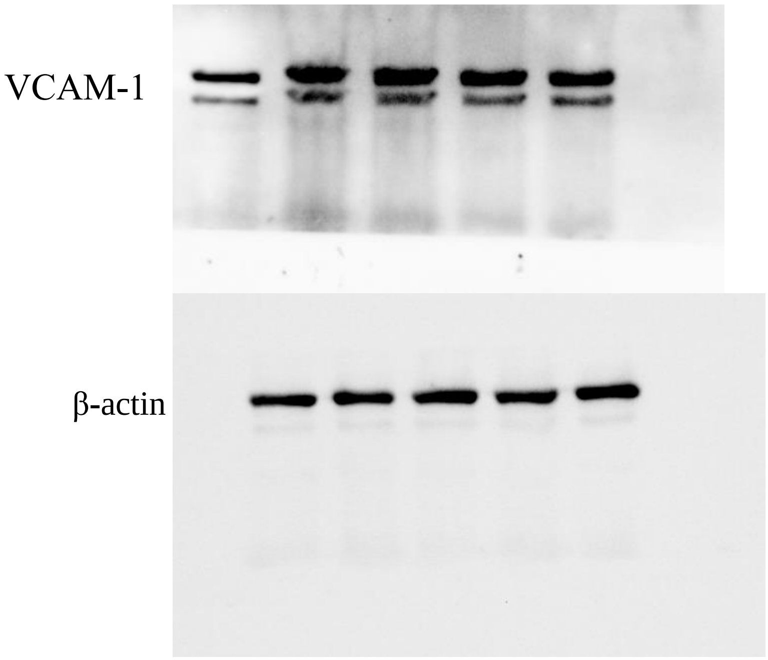

Fig C. CTRP3 inhibited HGHL induced VCAM-1 production in an AMPK dependent manner.

1

pAKT

AKT

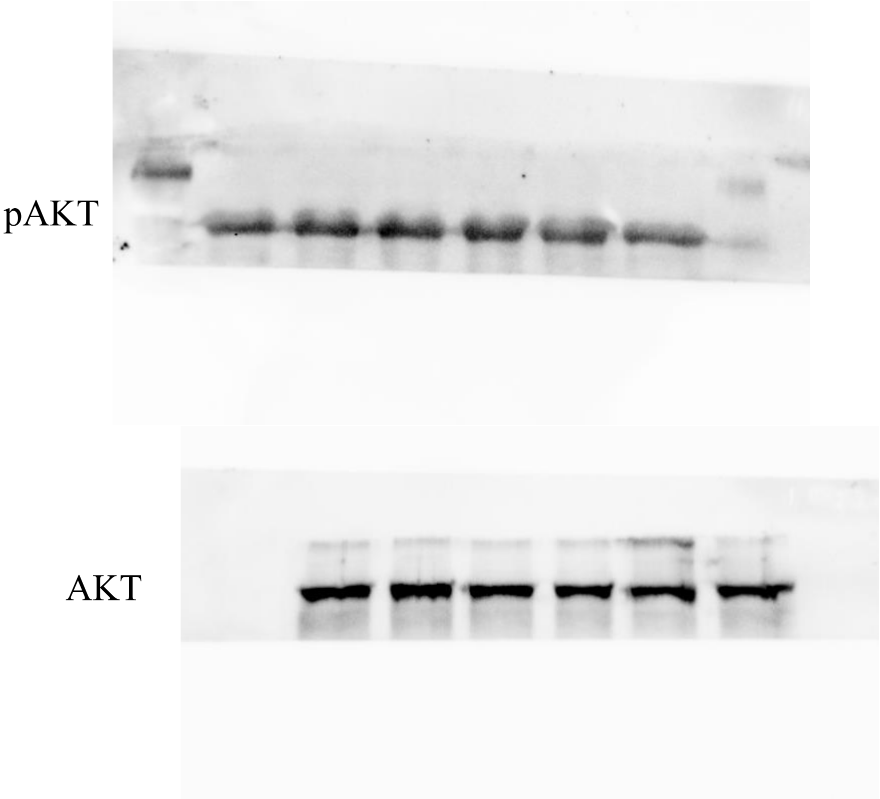

2

pAMPK

AMPK

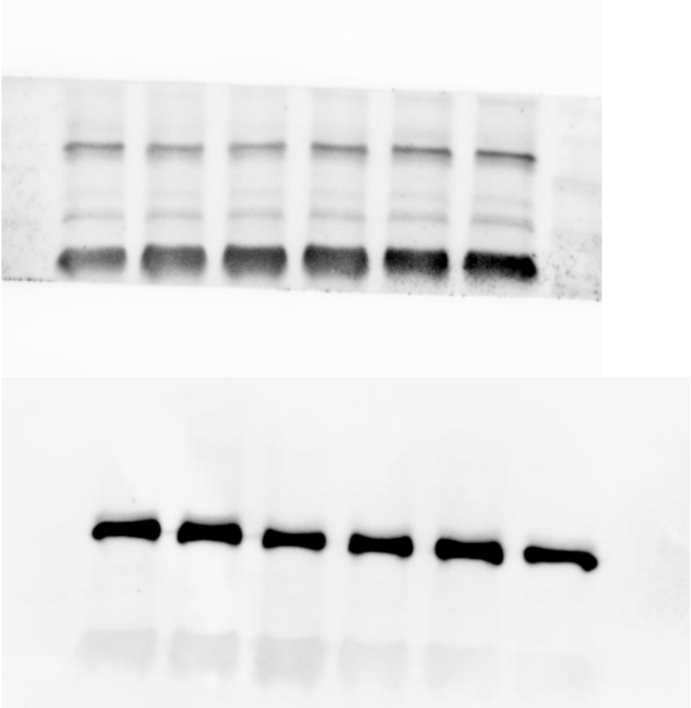

Fig C. 3

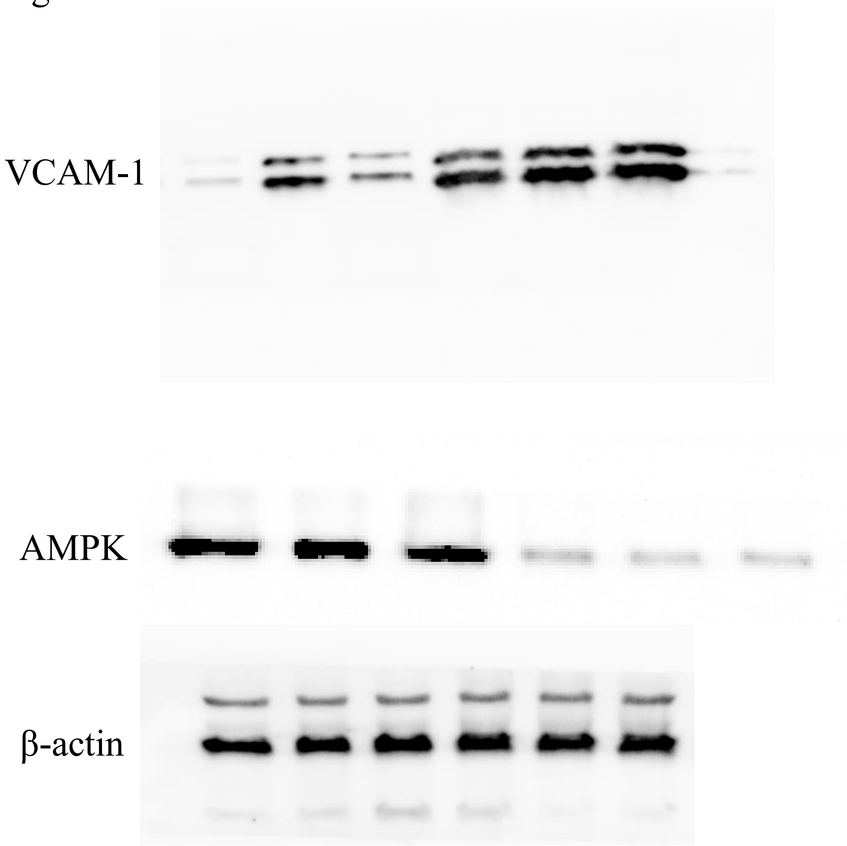

Supplement: S1 File — Table A. General characteristics of study subjects. Table B. Correlation analysis between DR and laboratory characteristics. Table C. Correlation between DR and CTRPs. Table D. Correlation analysis of variables associated with circulating CTRP3. All, n = 120. Table E. Correlation analysis of variables associated with circulating CTRP3. T2DM, n = 90. Table F. Correlation analysis of variables associated with circulating CTRP3. DR, n = 56. Fig A. ROC curves, for DR/PDR diagnosis, by circulating CTRP3 level. (1) ROC curve for DR diagnosis by circulating CTRP3 level. (2) ROC curve for PDR diagnosis by circulating CTRP3. Fig B. CTRP3 inhibits high glucose/high lipids (HGHL)-induced expression of VCAM-1 in a time- and dose-dependent manner. (1) VCAM-1 was inhibited in a concentration-dependent manner following CTRP3 administration. (2) VCAM-1 significantly decreased after 15 minutes post CTRP3 treatment. Fig C. CTRP3 inhibited HGHL induced VCAM-1 production in an AMPK dependent manner. (1) Akt phosphorylation was determined after different dose CTRP3 administration. (2) AMPK phosphorylation was determined after 48 hours of HGHL incubation, followed by CTRP3 treatment. (3) AMPK knockdown blocked CTRP3-mediated inhibition of VCAM-1 expression induced by HGHL. (PDF) [file pone.0178253.s001.pdf]
